# Cortical Thickness in Children Receiving Intensive Therapy for Idiopathic Apraxia of Speech

**DOI:** 10.1007/s10548-013-0308-8

**Published:** 2013-08-24

**Authors:** Darren S. Kadis, Debra Goshulak, Aravind Namasivayam, Margit Pukonen, Robert Kroll, Luc F. De Nil, Elizabeth W. Pang, Jason P. Lerch

**Affiliations:** 1 Division of Neurology, Pediatric Neuroimaging Research Consortium (PNRC), Cincinnati Children’s Hospital Medical Center, Cincinnati, OH USA; 2 College of Medicine, Pediatrics, University of Cincinnati, Cincinnati, OH USA; 3 The Speech and Stuttering Institute, Toronto, ON Canada; 4 Speech Language Pathology, University of Toronto, Toronto, ON Canada; 5 Department of Pediatrics, University of Toronto, Toronto, ON Canada; 6 Neurosciences and Mental Health, Hospital for Sick Children, Toronto, ON Canada; 7 Department of Medical Biophysics, University of Toronto, Toronto, ON Canada; 8 Neurology, Hospital for Sick Children, 555 University Avenue, Toronto, ON M5G 1X8 Canada

**Keywords:** Motor speech disorder, Childhood apraxia of speech (CAS), Plasticity, Supramarginal, Wernicke, MRI

## Abstract

Children with idiopathic apraxia experience difficulties planning the movements necessary for intelligible speech. There is increasing evidence that targeted early interventions, such as Prompts for Restructuring Oral Muscular Phonetic Targets (PROMPT), can be effective in treating these disorders. In this study, we investigate possible cortical thickness correlates of idiopathic apraxia of speech in childhood, and changes associated with participation in an 8-week block of PROMPT therapy. We found that children with idiopathic apraxia (*n* = 11), aged 3–6 years, had significantly thicker left supramarginal gyri than a group of typically-developing age-matched controls (*n* = 11), *t*(20) = 2.84, *p* ≤ 0.05. Over the course of therapy, the children with apraxia (*n* = 9) experienced significant thinning of the left posterior superior temporal gyrus (canonical Wernicke’s area), *t*(8) = 2.42, *p* ≤ 0.05. This is the first study to demonstrate experience-dependent structural plasticity in children receiving therapy for speech sound disorders.

**Electronic supplementary material:**

The online version of this article (doi:10.1007/s10548-013-0308-8) contains supplementary material, which is available to authorized users.

Idiopathic apraxia, a form of Speech Sound Disorder (SSD), is broadly characterized as deficits in the planning of movements necessary for intelligible speech. Children with apraxia of speech have difficulty producing target phonemes, either in isolation or succession, and typically present with inaccurate word production and dysfluency. Childhood apraxias emerge in the first years of life, and have the potential to negatively impact later language, academic, and social skills development. Unlike dysarthria, which is generally considered a neuromuscular disorder, apraxia may or may not be associated with neurological insult (see, American Speech-Language-Hearing Association 2007a, b, for a discussion on childhood apraxia of speech, CAS, and possible presentations and diagnostic challenges). In their recent literature review (Liegeois and Morgan 2012) found no evidence for unilateral lesions leading to apraxia in children; in the few cases of neurological insult associated with CAS, bilateral abnormalities in brain regions known to support language processing (i.e., perisylvian cortex) and motor control (basal ganglia, Rolandic cortex) were documented. Idiopathic apraxia of speech is among the most common SSD affecting children, yet it remains poorly understood in terms of etiology and stability.

There is mounting evidence that developmentally-appropriate targeted interventions can be effective in treating childhood SSDs (Tyler 2008; see also, Gierut 1998; Law et al. 2004). One approach, known as Prompts for Restructuring Oral Muscular Phonetic Targets (PROMPT), has been shown to be effective in treating CAS and other motor speech disorders (Chumpelik 1984; Grigos et al. 2010; Hayden 2006; Namasivayam et al. 2013). During PROMPT sessions, therapists provide direct tactile and kinesthetic cues, in addition to auditory and visual cues, to promote correct speech production. Clients practice requisite oral-motor positions and trajectories in words and phrases, gaining experience and familiarity with target sound production. Although researchers have begun to document the efficacy of targeted interventions such as PROMPT (e.g., Chumpelik 1984; Grigos et al. 2010; Hayden 2006; Namasivayam et al. 2013), the mechanisms for change and the neural correlates of participation in therapy remain unknown.

Recent advances in MRI technology and analyses permit quantitative and objective study of gross brain structure, which can be used to compare groups or to characterize neuroanatomical change over time. In their seminal study, (Maguire et al. 2000) found that London taxi drivers had larger posterior and smaller anterior hippocampi than age-matched controls, a difference thought to reflect the relative navigational experience of the two groups. Indeed, regional differences were associated with time spent driving a taxi, providing compelling evidence for experience-dependent structural plasticity in the mature hippocampus. Others have shown that experience-dependent structural changes are not limited to deep structures. Using voxel-based morphometry (VBM), (Draganski et al. 2004) documented increased grey matter density in posterior temporal and inferior parietal regions in a group of adults learning to juggle. Cortical growth, although transient, occurred in regions known to support visual processing of moving objects. Using similar approaches, others have documented visual spatial or navigational training-induced effects on cortical structure (Ilg et al. 2008; Wenger et al. 2012).

Increasingly, researchers are documenting training-induced structural changes in pediatric populations. Hyde et al. (2009) used deformation-based morphometry (DBM) to compare the brains of healthy children, 6 years of age, who received instrumental musical training with a cohort who did not. At baseline (prior to first music lessons), no structural differences were observed. Over the course of 15 months, however, both cortical and subcortical effects emerged: the group receiving musical training showed morphometric differences in several areas, including increased volume in the right precentral gyrus, right Heschl’s gyrus, and a mid region of the corpus callosum, consistent with previous reports of structural differences in musicians and non-musicians. Changes in cortical regions were correlated with performance on motor and melodic/rhythmic discrimination tasks, supporting the argument that musical training can drive structural change. Others have documented cortical volume changes in children receiving behavioral interventions for ADHD (Hoekzema et al. 2011), and dyslexia (Krafnik et al. 2011; see also Gebauer et al. 2012; Keller and Just 2009). Quantitative neuroanatomical investigations suggest a massive potential for structural plasticity in pediatric therapeutic contexts.

The goals of this study are to (1) assess cortical thickness correlates of idiopathic verbal apraxia in childhood, and (2) characterize changes in cortical thickness associated with participation in PROMPT therapy. We chose vertex-based thickness analyses over conventional VBM and DBM, as the approach shows relative sensitivity to subtle cortical effects (see Hutton et al. 2009; Scanlon et al. 2011). To assess structural correlates of the disorder and intervention, we collected high-resolution MR images from 14 young children referred for treatment of verbal apraxia, and a group of 14 typically developing controls. The apraxia group then received 8 weeks of PROMPT therapy, before returning for follow-up scans. The children with apraxia had presumably idiopathic disorders; however, in the absence of obvious structural abnormalities or lesions, we expect subtle cortical differences in regions known to support speech and language (see Liegeois and Morgan 2012). In children receiving therapy, we expect gains to be reflected in cortical thickness change in canonical language areas and sensory-motor regions (i.e., perisylvian and Rolandic cortices).

## Method

### Participants

Fourteen children with idiopathic apraxia of speech, and 14 typically developing children (Controls) participated in this study. Children with apraxia were recruited from a large pool of children referred to The Speech and Stuttering Institute (Toronto, ON, Canada) for treatment of speech sound disorders. The apraxia group consisted of 9 males and 5 females, aged 3.9–6.6 years (M = 4.5, SD = 0.8) with confirmed moderate to severe speech difficulties, characterized by focal motor planning deficits (apraxia), in the absence of known neurological disorders, neuromuscular deficits (dysarthria), or hearing problems. Children in the apraxia group completed baseline, intervention, and follow-up components of the study over a 10-week period. Baseline testing involved brief cognitive assessment, comprehensive speech language assessment, and neuroimaging. Within 1 week of completing baseline assessments, the apraxia group began an 8-week block (16 sessions total) of PROMPT therapy (in all cases, therapy was provided without charge to children and their families). Within 1 week of completing PROMPT, participants with apraxia completed a repeat speech language assessment and neuroimaging.

The Control group consisted of 8 males and 6 females, 4.1–6.3 years of age (M = 4.1, SD = 0.7). Controls were recruited from the community, and were negative for history of developmental delay, neurological disorder, hearing problems, and speech language impairment. Controls underwent baseline cognitive assessment and neuroimaging, but did not receive speech assessments or any speech training. All Controls were invited to return for repeat neuroimaging after 10 weeks; however, only a small subset (*n* = 4) were available at the required interval. Demographic information for both groups is presented in Table 1.

**Table 1:** Demographic and neuropsychological profile of participants

	Apraxia mean (SD)	Control mean (SD)	Between-groups *t*-test
Age in years	4.54 (0.83)	4.95 (0.72)	*t*(26) = 1.4, *p* > 0.05
Handedness			
EHI	52.53 (69.95)	60.43 (42.44)	*t*(26) = 0.4, *p* > 0.05
Receptive language			
PPVT—*z* score	0.27 (0.94)	1.16 (0.91)	*t*(26) = 2.5, *p* ≤ 0.05
Expressive Language			
EVT—*z* score	0.56 (0.78)	1.14 (0.88)	*t*(25) = 1.8, *p* > 0.05
Nonverbal Functioning			
WNV, 2-subtest FSIQ estimate, *z* score	−0.10 (1.17)	0.12 (1.09)	*t*(26) = 1.7, *p* > 0.05

*EHI* Edinburgh handedness inventory, *PPVT* peabody picture vocabulary test, *EVT* expressive vocabulary test, *WNV* Wechsler nonverbal scale of intelligence

Speech assessments and therapy were conducted at The Speech and Stuttering Institute; cognitive assessments and neuroimaging were carried out at the Hospital for Sick Children (Toronto, ON). The study was approved by the Hospital’s Research Ethics Board.

## Procedure

### Cognitive Assessment

To estimate gross cognitive functioning, both groups underwent brief baseline assessment with the Peabody Picture Vocabulary Test (PPVT; Dunn and Dunn 2007), Expressive Vocabulary Test (EVT; Williams 2007), and the Wechsler Nonverbal Scale of Ability (WNV; Wechsler and Naglieri 2006). These are standardized tests of receptive language, expressive language, and nonverbal functioning, respectively. Both groups scored within normal limits across all measures (see Table 1); for the apraxia group, intact gross language functioning suggests a focality of speech-motor deficit.

### Prompts for Restructuring Oral Muscular Phonetic Targets therapy

Only children with apraxia received speech therapy. The PROMPT approach has been documented previously (Chumpelik 1984; Grigos et al. 2010; Hayden 2006; Namasivayam et al. 2013), and is described here only briefly. Therapists first identify target areas for remediation and then develop individualized programs to address each client’s specific speech production errors. In all cases, therapy involves direct tactile-kinesthetic cuing applied to the mouth and face; cues inform of correct positions and movement trajectories, thus promoting correct articulation and fluency. Tactile-kinesthetic cues are supported by visual cues and auditory models along with verbal feedback on the quality and success of speech attempts, collectively forming a comprehensive multi-sensory intervention. For example, if while attempting to produce the word “pop”, the child exhibited excess jaw excursion and lateral sliding, the clinician would instruct the child to use a small mouth opening and keep their jaw in midline; the clinician would model production of the word “pop”, and then guide the child’s mandibular movement using their thumb and index finger while the child repeatedly attempts “pop” production. In this study, PROMPT was provided by the same therapist (author DG); although participants present with variable speech production errors, therapy followed a consistent routine (Namasivayam et al. 2013), with the common goal of promoting development of new and stable motor programs. Between PROMPT sessions, the children worked with their parents to practice prescribed daily home-based activities for approximately 5–10 min per day (e.g., parent reminds child to keep mouth opening small and jaw in midline while producing the word “pop”, and then plays game involving blowing and catching bubbles and saying “pop”).

### Speech Assessment

Children in the apraxia group underwent comprehensive speech assessments before and after speech therapy. We report on performance on three widely-used standardized devices (see below). Assessments were videotaped and speech samples collected (16-bit 44.1 kHz recordings) for analyses. Performance of individuals contributing both pre- and post-intervention scans is presented in Supplementary Table 1.

#### Goldman-Fristoe Test of Articulation 2 (GFTA-2; Goldman and Fristoe 2000)

The GFTA-2 is used to assess articulation in English speakers between the ages of 2 and 21 years. Word production is analyzed in initial, medial, and final positions, along with consonant blends in the initial position. For this study, we report on performance on the Sounds-in-Words subtest; raw scores are analyzed for therapy-induced change.

#### Hodson Computerized Analysis of Phonological Patterns (HCAPP; Hodson 2003)

The HCAPP is used to systematically measure phonological deviations in speech. Using 50 target words, errors are quantified along several dimensions. We report on Total Occurrence of Major Phonological Deviations.

#### Verbal Motor Production Assessment for Children (VMPAC; Hayden and Square 1999)

The VMPAC tests neuromotor integrity for speech in children ages 3–12 years. Scores on the Focal Oromotor Control and Sequencing subtests are reported as percentage correct values.

### MRI Acquisition and Processing

T1-weighted magnetization prepared rapid gradient echo (MPRAGE) images (sagittal, 3D; TR/TE/TI = 2300/2.96/900 ms, respectively; flip angle = 9°) were acquired at 1.0 × 1.0 × 1.0 mm resolution using a Siemens Trio 3T scanner (Siemens Aktiengesellschaft, Munich, Germany). Images were submitted to CIVET version 1.1.10, an automated processing pipeline, involving non-uniformity correction (Sled et al. 1998), stereotactic registration (Collins et al. 1994), skull stripping (Smith 2002), and tissue classification (Tohka et al. 2004). Inner and outer cortical surfaces were extracted for vertex-based analyses (Kim et al. 2005; Lerch and Evans 2005). Cortical thickness was calculated as the difference between linked inner and outer surface vertices (40,962 pairs per hemisphere), smoothed using a 20 mm surface-based kernel (Lerch and Evans 2005).

### Regions-of-Interest (ROIs)

To minimize the number of comparisons, we confined our analyses to areas known to support language, speech, and voluntary oral-motor control. We developed discreet ROIs for canonical Broca’s area (left *pars opercularis*) and Wernicke’s area (the superior temporal gyrus posterior to Heschl’s convolutions) and their right hemisphere homologues, neighboring gyri of the frontal and temporal lobes, and inferior aspects of the pre- and post-central gyri. Because the temporal-parietal junction is frequently included as an extracanonical region contributing to Wernicke’s area (see Bogen and Bogen 1976), we also developed bilateral ROIs for the posterior half of the supramarginal gyrus, inferior to the ascending ramus of the Sylvian fissure. See Supplementary Fig. 1 online. ROIs were drawn, extending to the depths of sulci, on an average surface of all subjects’ scans after non-linear surface-based registration (Lyttelton et al. 2007). Cortical thickness maps, computed in native space for each scan, were then brought into average space using the same surface deformation field. The transformed thickness maps were then used to compute mean thickness within each ROI for each subject, for each scan visit. In order to minimize variability and focus on local effects, cortical thickness maps were scaled by dividing the thickness at each vertex pair by the mean thickness for the respective hemisphere; mean scaled thickness for each ROI was used in both between-groups and within-subjects analyses.

### Statistical Analyses

To assess correlates of the disorder, we compared the apraxia and Control groups for baseline scaled cortical thickness at each ROI using independent samples *t*-tests. For any region showing significant group differences, we compared baseline speech performance (GFTA-2, HCAPP, and VMPAC scales) to scaled cortical thickness in the apraxia group using bivariate Pearson correlations. To assess correlates of therapy, we analyzed pre- to post-intervention thickness changes occurring at each ROI in the clinical group using within-subjects *t*-tests. For any region showing significant within-subjects effects, we compared changes in speech performance (GFTA-2, HCAPP, and VMPAC scales) to changes in scaled cortical thickness using bivariate Pearson correlations, and describe changes occurring in the small subset of Controls who were scanned serially. Due to the limited sample and exploratory nature of this study, we did not correct for multiple comparisons across ROIs.

## Results

### Efficacy of PROMPT Therapy

In all cases, parents of children with idiopathic verbal apraxia reported that PROMPT therapy was beneficial for their children. Two children in the apraxia group did not return for follow-up speech assessment or MRI, due to anxiety experienced during baseline assessment; we report on the remaining 12 children with apraxia, who showed significant (*p* < 0.05) gains on all speech measures. Change scores and within-subjects statistics are presented in Table 2.

**Table 2:** Changes in speech performance following PROMPT therapy

Measure	Magnitude of mean difference	Within-subjects *t*-test
GFTA-2 sounds-in-words—raw score	9.1	*t*(11) = 3.6, *p* = 0.004
HCAPP Phonological deviations—total	39.7	*t*(11) = 7.3, *p* ≤ 0.001
VMPAC focal motor control—% correct	11.5	*t*(11) = 3.7, *p* = 0.004
VMPAC sequencing—% correct	7.7	*t*(11) = 3.1, *p* = 0.010

*GFTA* Goldman Fristoe test of articulation, *VMPAC* Verbal motor production assessment for children, *HCAPP* Hodson computerized analysis of phonological patterns

### Cortical Thickness Correlates of Apraxia

Of the 28 children in this study, 11 children with apraxia (8 males, mean age 4.7 years) and 11 Controls (5 males, mean age 4.8 years) had baseline MRIs suitable for analyses (i.e., with cortical morphology retained after segmentation; rejected scans showed obvious movement-related artifact). Suitability was determined by agreement of two independent raters, blind to group membership.

**Fig. 1:**
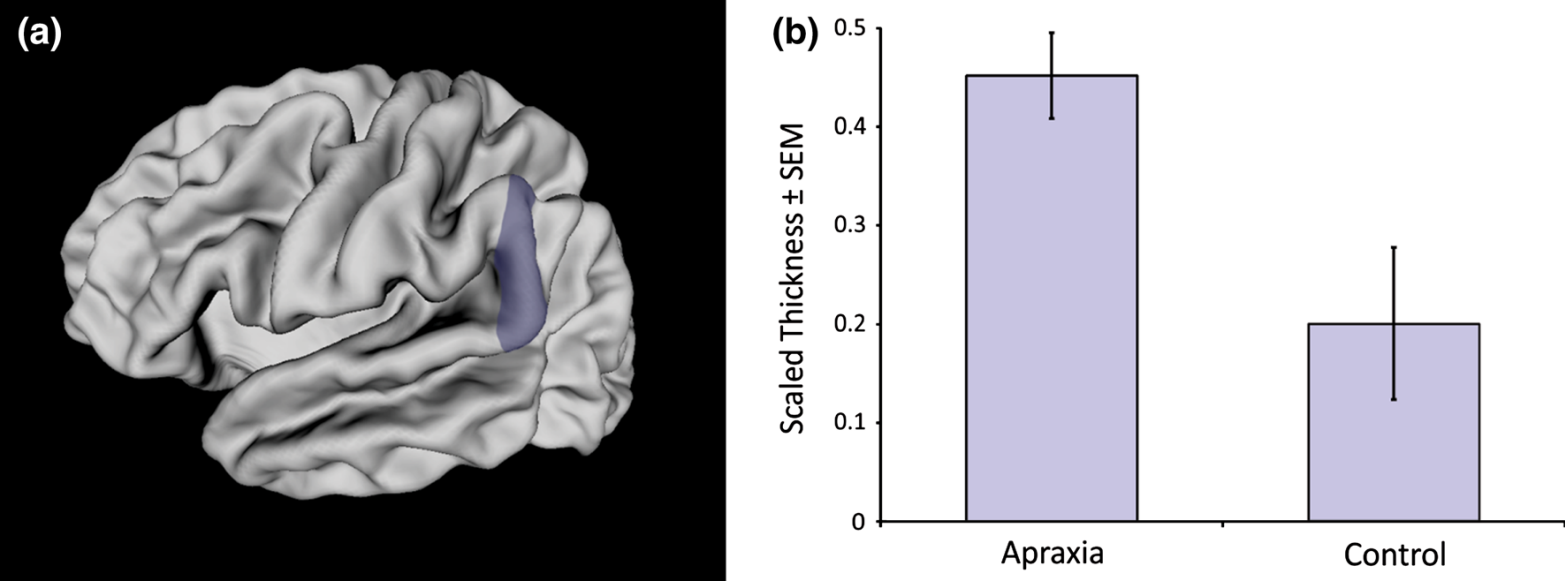
**a** Left posterior supramarginal gyrus ROI, represented as a *shaded region* on a mid-surface rendering of an average brain; **b** children with idiopathic apraxia (*n* = 11) had thicker left posterior supramarginal gyri compared to Controls (*n* = 11) at baseline, *t*(20) = 2.84, *p* ≤ 0.05. *Mean scaled cortical thickness* (±*SEM*), shown for each group (Color figure online)

No significant between-groups differences were observed for overall cortical thickness or mean thickness within each hemisphere (*t*-tests, *p* > 0.05). Only one significant between-groups difference in ROI analyses was observed: children with idiopathic apraxia had significantly thicker left posterior supramarginal gyri than Controls, *t*(20) = 2.84, *p* ≤ 0.05. See Fig. 1. Left posterior supramarginal gyrus thickness did not correlate with any of the baseline measures of speech performance in the clinical group, *p* > 0.05.

### Cortical Thickness Changes Following PROMPT

Nine children (6 male; mean age 4.5 years) with mild apraxia had pre- and post-intervention MRIs that were suitable for cortical thickness analyses. Of the four Controls studied at follow-up, three (2 male; mean age 5.5) had suitable baseline and follow-up scans; due to the small number with repeat neuroimaging, we excluded Controls from formal longitudinal analyses, and provide only a description of change for informal comparison with the group receiving intervention.

**Fig. 2:**
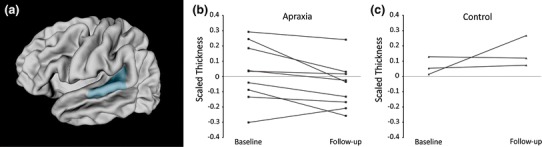
**a** Left posterior superior temporal gyrus (Wernicke’s area), represented as *shaded region*; **b** children with idiopathic apraxia (*n* = 9) experienced significant thinning of Wernicke’s over the course of therapy, (*t*(8) = 2.42, *p* ≤ 0.05); **c** baseline and follow-up scaled cortical thickness of Wernicke’s area in the small subset of Controls with appropriate serial imaging (*n* = 3) (Color figure online)

In children receiving therapy for apraxia, ROI analyses revealed significant thinning in the posterior superior temporal gyrus, canonical Wernicke’s area, *t*(8) = 2.42, *p* ≤ 0.05. See Fig. 2. While 8 of the 9 children with apraxia showed thinning in Wernicke’s following therapy, only 1 of the 3 Controls showed thinning over the same period. Decreasing thickness in Wernicke’s over the course of therapy was not significantly correlated to change scores on any of the standardized speech measures, *p* > 0.05.

## Discussion

This is the first study to investigate cortical thickness correlates of idiopathic apraxia of speech and changes in cortical thickness associated with brief intensive speech therapy in children. We found that children with apraxia had thicker left supramarginal gyri than Controls. In the absence of appreciable lesions, the quantitative neuroanatomical approach reveals a subtle morphological atypicality associated with motor speech deficits. The study also provides support for the benefits of PROMPT and speech motor intervention in children. Over the course of therapy, children with apraxia experienced thinning of the left posterior superior temporal gyrus (Wernicke’s area). This focal change evidences a potential for rapid and robust experience-dependent structural plasticity in childhood (see also, Hoekzema et al. 2011, Hyde et al. 2009; Krafnik et al. 2011).

The clinical significance of thicker left supramarginal gyri in children with idiopathic apraxia is not clear. The effect is apparent only at the group level, and thickness in the region does not correlate to degree of speech impairment. A thicker left supramarginal gyrus may reflect a subtle pathology; if sustained throughout childhood, a thick left supramarginal gyrus may indicate immaturity or altered development, as the region is expected to overgrow in the first years of life and prune back in childhood and early adolescence (Shaw et al. 2008; Sowell et al. 2004; see also, Porter et al. 2011). In adults, the left supramarginal gyrus is known to play an important role in speech production; injury to this region is associated with an apraxic presentation characterized by phonemic discrimination and speech planning deficits (for a recent review, see Gow 2012). However, the relationship between left supramarginal integrity and speech production in childhood has not been established. In their recent literature review, (Liegeois and Morgan 2012) found that childhood apraxia of speech and dysarthria were associated with bilateral, but not unilateral perisylvian insult. Our findings suggest that subtle unilateral atypicalities may underlie observed speech deficits in the absence of neurological injury.

Perhaps the most interesting finding in this study is that children with apraxia in our study experienced significant thinning in the posterior superior temporal gyrus, canonical Wernicke’s area, after only 8 weeks of PROMPT therapy. To our knowledge, this is the first study to demonstrate experience-dependent structural plasticity in children with speech sound disorders. Although the amount of cortical thinning was not significantly correlated to performance change on the standardized speech measures, 8 of 9 children in the apraxia group demonstrated thinning of Wernicke’s, and all experienced speech gains. The small subset of Controls with serial imaging showed a tendency toward increasing thickness (2 of 3 participants) in the same area, over the same period. Given the brief interval between scans and the spatial specificity of changes, it is likely that participation in the therapy program drove the observed cortical thinning in the clinical group. With a longer block of therapy, we may expect more robust changes and/or involvement of additional regions, particularly those known to support oral-motor control and expressive language (i.e., Rolandic and inferior frontal cortex).

The location of between- and within-group effects speaks to the deficits observed in idiopathic apraxia of speech, and a possible mechanism for gains enjoyed by participants receiving PROMPT. The left supramarginal gyrus and neighboring posterior superior temporal gyrus are each involved in sensorimotor integration, and are necessary for accurate speech comprehension and production (Gow 2012). Children with motor speech disorders are known to have deficits in phonological processes (McNeill et al. 2009; see also, Tkach et al. 2011), which are indirectly addressed in PROMPT therapy. During each session, PROMPT therapists provide tactile and kinesthetic cues to help clients produce target phonemes. Feedback is provided externally by the therapist (i.e., with touch and speech, with the provision of auditory models), and internally through somatosensory and auditory information. Collectively, the multimodal and multisensory feedback serves to reinforce the training. In PROMPT, development of phonological processing skills provides an internal validator for subsequent articulation attempts. Wernicke’s area undergoes thinning (possibly neuronal pruning) during participation in PROMPT therapy, which may reflect the rapid development of sensorimotor processes necessary for accurate speech production.

One of the major challenges in conducting this sort of research is obtaining homogenous clinical samples that are sufficiently large for the study of subtle structural effects. To minimize the number of comparisons conducted, we confined our analyses to a small set of ROIs established on theoretical grounds. We observed both between-groups and within-subjects effects with modest samples, demonstrating a sensitivity of the quantitative neuroanatomical approach; however, we may have lacked sufficient power to fully characterize the structural correlates of idiopathic verbal apraxia and PROMPT therapy. In the future, large-scale studies of children with apraxia will permit whole-brain analyses, potentially revealing relatively subtle or focal correlates of the disorder or of therapy that are not easily detected in relatively coarse ROI analyses. The inclusion of protracted follow-up scans and behavioral assessments will speak to the stability and functional relevance of the documented short-term changes. Future studies will also benefit from the inclusion of a comparably sized control group that is scanned serially. Longitudinal study of control participants is required for distinction of normal developmental changes and those occurring as a result of intervention. This is particularly the case when investigating structural change over long intervals (i.e., with sustained long-term speech therapy), as the pediatric brain is known to undergo substantial morphological change as part of normal development.

The quantitative neuroanatomical approach described in this study can be easily implemented with other populations, including children with complex or neurogenic forms of apraxia or dysarthria. Group analyses permit assessment at a much finer scale than possible through reading of individual scans. Obtaining high-quality MRIs of young children without sedation can be challenging, but is worth attempting, particularly for idiopathic disorders. There is some evidence that the potential for experience-dependent structural change decreases with advancing age (Wenger et al. 2012); the study of pediatric populations over time, or over the course of therapy, may be of relatively high yield. In the current study, serial assessment of a modest sample of children receiving PROMPT for idiopathic apraxia informed of a possible mechanism and neural target for therapeutic action—the intervention promoted development of sensory-motor systems controlling speech production, associated with thinning and possible maturation of Wernicke’s area.

## Electronic supplementary material

Below is the link to the electronic supplementary material.

Supplementary material 1 (DOCX 19 kb)

Supplementary material 2 (TIF 676 kb)
