# Supplementary material for: Cortical Thickness in Children Receiving Intensive Therapy for Idiopathic Apraxia of Speech
Source: Brain Topogr. 2013 Aug 24;27(2):240–7. doi: 10.1007/s10548-013-0308-8 (PMC3921462; doi:10.1007/s10548-013-0308-8)
Supplement: Supplementary file 1 — Supplementary material 1 (DOCX 19 kb) [file 10548_2013_308_MOESM1_ESM.docx]

Supplementary Table 1. *Speech performance before (Pre) and after (Post) PROMPT therapy in participants with idiopathic apraxia.*

| Age at Enrolment |  | Pre GFTA | Post GFTA |  | Pre HCAPP Total | Post HCAPP Total |  | Pre VMPAC Focal | Post VMPAC Focal |  | Pre VMPAC Sequencing | Post VMPAC Sequencing |
| --- | --- | --- | --- | --- | --- | --- | --- | --- | --- | --- | --- | --- |
|  |  |  |  |  |  |  |  |  |  |  |  |  |
| 4.0 |  | 49 | 46 |  | 106 | 82 |  | 170 | 189 |  | 25 | 28 |
| 4.0 |  | 54 | 28 |  | 130 | 64 |  | 134 | 164 |  | 14 | 22 |
| 4.0 |  | 39 | 38 |  | 90 | 67 |  | 184 | 222 |  | 29 | 38 |
| 4.0 |  | 47 | 47 |  | 112 | 92 |  | 205 | 210 |  | 34 | 37 |
| 4.2 |  | 50 | 47 |  | 102 | 78 |  | 151 | 188 |  | 27 | 27 |
| 4.3 |  | 26 | 11 |  | 65 | 15 |  | 201 | 208 |  | 34 | 37 |
| 4.4 |  | 44 | 27 |  | 94 | 32 |  | 222 | 222 |  | 34 | 34 |
| 4.5 |  | 40 | 26 |  | 55 | 32 |  | 209 | 217 |  | 33 | 37 |
| 4.6 |  | 47 | 36 |  | 117 | 75 |  | 147 | 184 |  | 22 | 32 |
| 4.8 |  | 66 | 65 |  | 214 | 163 |  | 39 | 160 |  | 18 | 14 |
| 6.2 |  | 42 | 42 |  | 84 | 61 |  | 196 | 224 |  | 28 | 32 |
| 6.6 |  | 48 | 30 |  | 104 | 36 |  | 180 | 208 |  | 39 | 40 |

Age at enrolment in years; GFTA = Goldman Fristoe Test of Articulation, raw scores; HCAPP = Hodson Computerized Analysis of Phonological Patterns, Total Occurrence of Major Phonological Deviations; VMPAC = Verbal Motor Production Assessment for Children, raw scores for Focal Oral Motor Control and Sequencing subscales
