# Supplementary figures and images for: Cortical Thickness in Children Receiving Intensive Therapy for Idiopathic Apraxia of Speech
Source: Brain Topogr. 2013 Aug 24;27(2):240–7. doi: 10.1007/s10548-013-0308-8 (PMC3921462; doi:10.1007/s10548-013-0308-8)

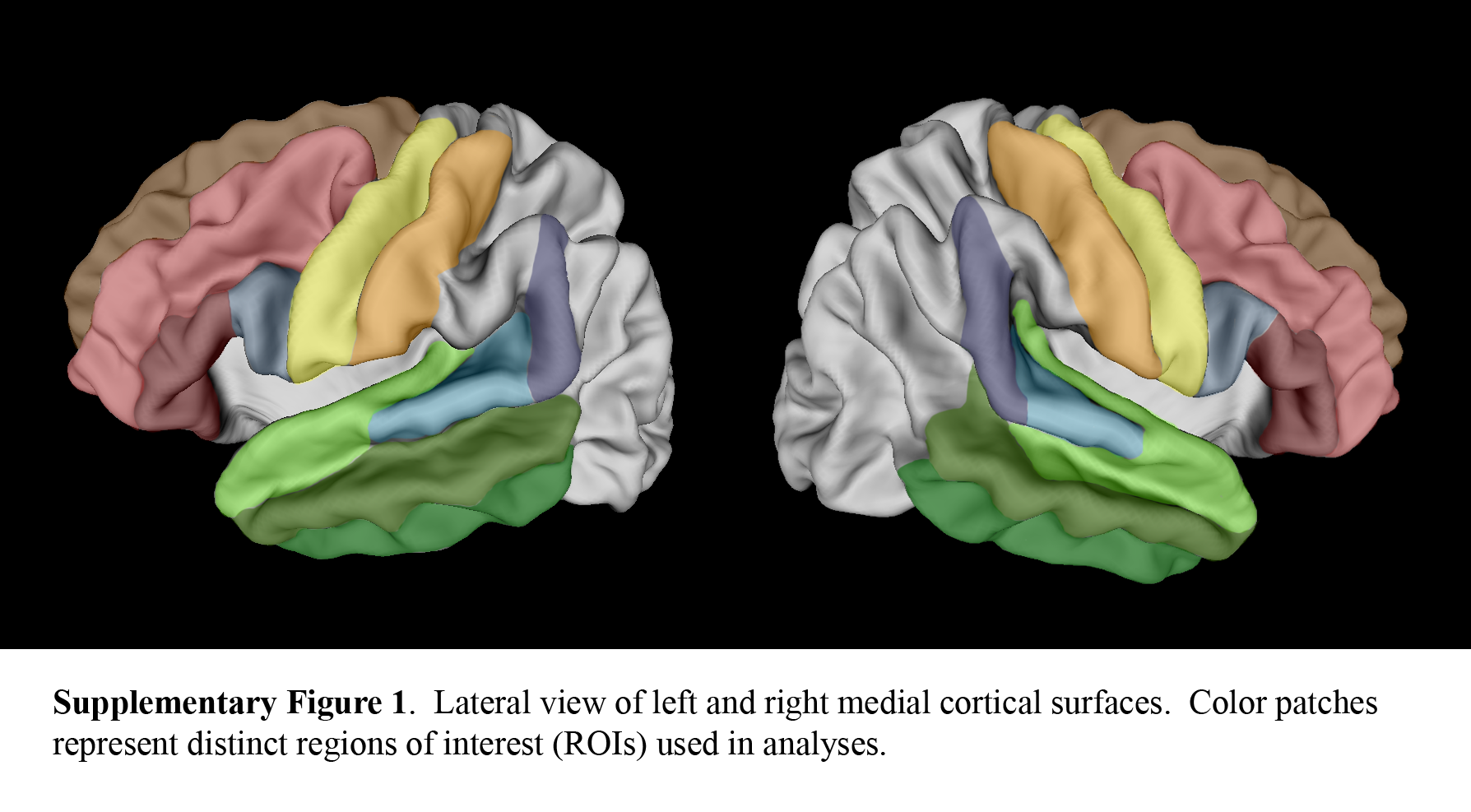

Supplement: Supplementary file 2 — Supplementary material 2 (TIF 676 kb) [file 10548_2013_308_MOESM2_ESM.tif]
